# PXR and CAR single nucleotide polymorphisms influence plasma efavirenz levels in South African HIV/AIDS patients

**DOI:** 10.1186/1471-2350-13-112

**Published:** 2012-11-22

**Authors:** Marelize Swart, Heather Whitehorn, Yuan Ren, Peter Smith, Rajkumar S Ramesar, Collet Dandara

**Affiliations:** 1 Division of Human Genetics, Faculty of Health Sciences, University of Cape Town, Observatory, Cape Town 7925, South Africa; 2 Department of Clinical Pharmacology, Faculty of Health Sciences, University of Cape Town, Observatory, Cape Town 7925, South Africa

**Keywords:** CAR, Efavirenz, HIV/AIDS, PXR, Pharmacogenetics, South Africa

## Abstract

**Background:**

This study investigated variation in *NR1I2* and *NR1I3* and its effect on plasma efavirenz levels in HIV/AIDS patients. Variability in plasma drug levels has largely led research on identifying causative variants in drug metabolising enzyme (DME) genes, with little focus on the nuclear receptor genes *NR1I2* and *NR1I3*, coding for PXR and CAR, respectively, that are involved in regulating DMEs.

**Methods:**

464 Bantu-speaking South Africans comprising of HIV/AIDS patients on efavirenz-based treatment (n=301) and 163 healthy subjects were genotyped for 6 SNPs in *NR1I2* and *NR1I3*. 32 of the 301 patients had their DNA binding domains (DBDs) in *NR1I2* and *NR1I3* sequenced.

**Results:**

Significantly decreased efavirenz plasma concentrations were observed in patients carrying the *NR1I3 rs3003596C/C* and *T/C* genotypes (P=0.015 and P=0.010, respectively). Sequencing resulted in the discovery of a further 13 SNPs, 3 of which are novel variants in the DBD of *NR1I2*. There were significant differences in the distribution of *NR1I2* and *NR1I3* SNPs between South Africans when compared to Caucasian, Asian and Yoruba population groups.

**Conclusion:**

For the realisation of personalised medicine, PXR and CAR genetic variation should be taken into consideration because of their involvement in the regulation of DMEs.

## Background

The pregnane X receptor (PXR) and the constitutive androstane receptor (CAR) are members of the orphan nuclear receptor (NR) superfamily and function primarily as sensors of xenobiotics by up-regulating the expression of drug metabolising enzymes (DMEs), facilitating the elimination of xenobiotics from the body [1,2]. PXR and CAR are transcriptional regulators of a wide range of genes whose products metabolise a wide range of drugs [2,3].

PXR and CAR are localised in the cytoplasm in their inactive state, bound to histone deacetylase complexes (HDACs). Upon ligand binding, PXR and CAR dissociate from the HDACs. PXR translocates into the nucleus where it heterodimerises with the retinoid X receptor (RXR), while CAR is nuclear translocated through a phosphorylation-dependent mechanism and subsequently heterodimerises with RXR [4]. In the nucleus, the heterodimers PXR/RXR and CAR/RXR bind to nuclear receptor response elements upstream of genes coding for DMEs) [4].

*NR1I2* gene, which encodes PXR, consists of 10 exons and is located on chromosome 3q13-21 [5]. The *NR1I2* DNA binding domain (DBD) is encoded by exons 3 and 4 whereas exons 5–10 code for the ligand binding domain (LBD). The DBD and LBD are separated by a hinge region encoded by a small portion of exon 5 [5]. Several SNPs have been reported in *NR1I2* and some are associated with changes in PXR function*.* For example, *NR1I2 63396C>T (rs2472677)*, which is located in a putative transcription factor binding site, has been associated with increased *NR1I2* expression in the presence of the *63396T* variant, which leads to increased *CYP3A4* expression leading to decreased atazanavir (ATV) plasma concentrations [6-8]. Three SNPs in exon 2 have been reported, namely *NR1I2 52G>A (E18K; rs59371185), 79C>T (P27S; rs12721613)* and *106G>A (G36R; rs12721607)*. The *NR1I2 79T* and *NR1I2 106A* alleles are associated with decreased *NR1I2* expression [5]. Another SNP, *NR1I2 7635A>G (rs6785049)*, is present in intron 5 and the *7635G* allele has been associated with increased expression of *CYP3A4* in the presence of rifampicin [9].

The *NR1I3* gene, which encodes CAR, is located on chromosome 1q21-23, and consists of 9 exons. Twenty-two unique *NR1I3* variants have been described where each isoform results from a different combination of splicing events. Some isoforms produce non-functional proteins due to the presence of nonsense mutations [10,11]. *NR1I3* isoform-3 has been suggested as the wild-type and produces a 348 amino acid protein [11]. The *NR1I3* DBD is encoded by exons 2, 3 and the 5’ portion of exon 4 [12]. Previously characterised SNPs in *NR1I3* include *NR1I3 rs2307424C>T*, of which the *rs2307424T* allele has been associated with low efavirenz plasma concentrations and the *NR1I3 rs2307424C/C* genotype has been associated with early discontinuation of efavirenz-containing anti-retroviral therapy (ART) in Caucasian HIV/AIDS patients [13].

Genetic characterization of indigenous African populations is slowly building up. This study aimed to further contribute to the genetic characterization of African populations by genotyping *NR1I2* and *NR1I3* and evaluating the effects of their variants on the response to efavirenz treatment in HIV/AIDS Bantu-speaking South African patients. In order to accelerate discovery of novel SNPs, the DBD of both *NR1I2* and *NR1I3* were targeted for sequencing [1].

## Methods

### Study subjects

The study cohort consisted of four-hundred and sixty-four (n=464) Bantu-speaking South Africans made up of healthy subjects (n=163) and HIV/AIDS patients (n=301) undergoing efavirenz-based treatment for at least six months. The subjects were recruited from Gauteng and Cape Town. Written informed consent was obtained and each participant provided demographic information such as 1) their ethnic group, 2) health status, 3) dietary habits, 4) smoking habits, and 5) home language were captured using a questionnaire. The study was approved by the Research Ethics Committee of the Faculty of Health Sciences at the University of Cape Town and the University of Witwatersrand Human Research Ethics Committee, Gauteng, South Africa and was performed in accordance with the guidelines of the Helsinki Declaration of 2008.

Two blood samples were obtained for DNA extraction and plasma efavirenz levels, respectively. DNA isolation was performed according to the method adapted from Gustafson *et al.,*[14] or the GenElute^TM^ Blood Genomic DNA Kit (Sigma-Aldrich, St. Louis, MO, USA) was used when blood sample volumes were limited. Steady state efavirenz plasma levels were available for 137 of the 301 HIV/AIDS patients and were collected 12–16 hours post-dose. Efavirenz concentrations were determined by the use of LC/MS/MS (API 4000 triple quadrupole MS/MS Applied Biosystems, South Africa) according to the method by Chi *et al.*, [15].

### Selection of SNPs and genotyping methods used

Three SNPs in *NR1I2* [GenBank: AF364606] and a further three SNPs in *NR1I3* [GenBank: BC069626.1] were investigated in this study. The six SNPs were selected based on previous reports of high minor allele frequencies in African-American and other African populations. SNPs were genotyped using either SNaPshot mini-sequencing or the PCR-RFLP method designed for *NR1I2 rs2472677C>T* (Additional file 1: Table S1).

PCR amplification was performed using the following conditions: initial denaturation at 94°C for 3 min, followed by 40 cycles of denaturation at 94°C for 30s, annealing at the specific temperature for each SNP for 30s, primer extension at 72°C for 20-45s depending on the primer sets and final extension at 72°C for 10 min. A “MyCycler Thermal cycler” (Bio-Rad, Hercules, USA) was used and the PCR reaction contained the following reagents; 50–100 ng of genomic DNA, 1X Green GoTaq Flexi Reaction Buffer (Promega Corporation, Madison, USA), 0.20 mM of each of the deoxynucleotide triphosphates (dNTPs) (Bioline, London, UK), 1.5 mM MgCl_2_ (Promega Corporation, Madison, USA), 40 pmol of the forward and reverse primers (Integrated DNA Technologies, Inc., Coralville, USA), 1U of GoTaq Flexi DNA Polymerase (Promega Corporation, Madison, USA). PCR amplification was followed by digestion using 3U *Hpy188I* (New England BioLabs, Inc., Ipswich) in the presence of 1X NEBuffer 4 (New England BioLabs, Inc., Ipswich) when genotyping for the *NR1I2 rs2472677C>T* polymorphism.

### SNaPshot multiplex

Five separate PCR amplification reactions were carried out. PCR products (5 μl) were then pooled for SNaPshot genotyping. The pooled PCR products were cleaned using 1.5U shrimp alkaline phosphatase (Fermentas Life Sciences, Burlington, Canada) and 2U *ExonucleaseI* (Fermentas Life Sciences, Burlington, Canada) to remove unincorporated primers and dNTPs. SNaPshot single base extension was performed using the “GeneAmp® PCR System 9700 version 3.08” (Applied Biosystems, Carlsbad, USA) under the following conditions; denaturation at 96°C for 10s, followed by 25 cycles of primer annealing at 50°C for 5s and primer extension at 60°C for 30s. To the 1 μl ABI Prism® SNaPshot™ Multiplex Kit (Applied Biosystems, California, USA), primers (Integrated DNA Technologies, Inc., Coralville, USA) for the pooled PCR products were added. The clean-up reaction was repeated using 1U shrimp alkaline phosphatase. An ABI 3130xl Genetic Analyzer (Applied Biosystems, Carlsbad, USA) was used for capillary electrophoresis and GeneMapper© Software version 4.1 (Applied Biosystems, Carlsbad, USA) was used to analyse results.

### Identification of novel SNPs

The *NR1I2* and *NR1I3* DNA binding domains (DBDs) were sequenced in 32 of the 301 HIV/AIDS patients to search for novel SNPs. The sequencing reaction used the ABI Prism® BigDye® Terminator Cycle Sequencing v3.1 Kit (Applied Biosystems, Carlsbad, CA, USA), which included 1 μl Terminator mix and 1X Sequencing buffer, together with the PCR fragment, and 1 μM of the forward or reverse primer. Analysis of the sequencing data was performed using BioEdit Sequence Alignment Editor v7.0.0. The novel SNPs were assessed for functional significance with the Functional Analysis of Novel SNPs (FANS) program ( http://genepipe.ncgm.sinica.edu.tw/fans/input.do) [16] and ESE finder v3.0.

### Statistical analysis

Statistical analyses were performed using the Graphpad Prism statistical program (Version 5, GraphPad Software Inc., San Diego, CA), Statistica v10.0 (StatSoft, USA) and Phase v2.1 [17-19]. Pearson’s *χ*^2^-test and Fisher’s exact test was used to compare the genotype and allele frequencies between the healthy participants and the HIV/AIDS patients as well as the allele frequencies in the South Africans to those of other populations with results in literature. The SHEsis statistical program was used for linkage disequilibrium (LD, D’ and r^2^) [20,21] analysis and Phase v2.1 for inferring of *NR1I2* and *NR1I3* haplotypes. Statistical significance was defined as *P* <0.05 and all statistical tests were performed two tailed.

## Results

### Demographic characteristics

The healthy subjects had a mean age of 35.8 years (SD ± 14.0 years), while the HIV/AIDS patients (consisting of 71 males, 226 females and 4 patients where gender were not recorded) had a mean age of 41.3 years (SD ±9.3 years). Among the HIV/AIDS patients, efavirenz plasma concentrations were available in 137 subjects. A summary of the baseline characteristics of the study cohort is outlined in Table 1. The efavirenz plasma concentration in the South African HIV/AIDS patients showed a large degree of variation (36-fold), ranging between 0.59 and 22 μg/mL, suggesting extensive inter-individual variability in efavirenz drug metabolism and disposition.

**Table 1:** Demographic characteristics of the healthy participants and HIV/AIDS patients

	**HIV/AIDS patients (n=301)**	
Gender ratio (Male: Female)	1:3.8	
Mean age, years (range)	41.3 ±9.3	(22–75)
Tobacco smoking		
Yes	20	(0.07)
No	268	(0.93)
Alcohol consumption		
Yes	29	(0.10)
No	259	(0.90)
Mean BMI, kg/m^2^ (range)	23.23 ±4.74	(11.7-40.6)
Average viral load at baseline, copies/ml (range)	26917.71 ±27133.50	(25–98400)
Average CD4 count at baseline, cells/μl (range)	136.09 ±113.24	(2–605)
Efavirenz-containing ARV regimens		
3TC_TDF_EFV	9	(0.03)
AZT_3TC_EFV	11	(0.04)
d4T_3TC_EFV	222	(0.74)
Average plasma efavirenz concentration, μg/mL (range)	4.64	(0.59 – 22)
Mean Haemoglobin level, IU/l (range)	11.58 ±2.14	(5.4-16.3)
Median AST level, IU/l (range)	32	(15 – 228)
Median ALT level, IU/l (range)	23.5	(6 – 176)
	**Healthy subjects (n=163)**	
Gender ratio (Male: Female)	1:2.3	
Mean age, years (range)	35.8 ±13.96	(21–63)
Tobacco smoking		
Yes	8	(0.15)
No	44	(0.85)
Alcohol consumption		
Yes	22	(0.42)
No	30	(0.58)

### Genotype frequencies

Genotype frequencies were compared between the healthy subjects and HIV/AIDS patients for the six SNPs, three each in *NR1I2* and *NR1I3,* genotyped using SNaPshot or PCR-RFLP. The genotypes of the healthy subjects were all in HWE (P>0.05) for the six SNPs. However, the *NR1I2 rs3732356T>G* (P=0.020) genotype frequencies deviated from HWE in the HIV/AIDS patients. Polymorphic variation was observed in all six SNPs and all genotypes were observed in both healthy subjects and HIV/AIDS patients except for the *NR1I2 rs6785049A/A* genotype, which was absent in the HIV/AIDS patients and the *NR1I3 rs2307424T/T* genotype, which was not observed in both the healthy subjects and HIV/AIDS patients (Table 2). The distribution of *NR1I2 rs3732356T>G* and *NR1I2 rs6785049G>A* genotypes were significantly different between the healthy subjects and HIV/AIDS patients (P=0.031 and 0.002, respectively) (Table 2). Although gender differences are known to result in differences in drug disposition, no association of gender with plasma efavirenz levels were observed in this study with average plasma efavirenz levels of 5.25 μg/mL and 4.43 μg/mL in males and females, respectively (P=0.307).

**Table 2:** Comparison of the genotype frequencies between control subjects and HIV/AIDS patients

**SNP**	**Genotype**	**Location/functional effect**	**Healthy subjects**	**HIV/AIDS patients**	**Global p-value**
			**n (freq)**	**n (freq)**	
NR1I2 rs3732356T>G	T/T	Intron 3	95 (0.605)	162 (0.549)	0.031
	T/G		50 (0.318)	123 (0.417)	
	G/G		12 (0.076)	10 (0.034)	
NR1I2 rs2472677C>T	C/C	Intron 1, Disrupts HNF3B TFB site	57 (0.368)	97 (0.365)	0.917
	C/T		75 (0.484)	133 (0.500)	
	T/T		23 (0.148)	36 (0.135)	
NR1I2 rs6785049G>A	G/G	Intron 5	128 (0.853)	264 (0.950)	0.002
	G/A		21 (0.140)	14 (0.050)	
	A/A		1 (0.007)	0 (0.000)	
NR1I3 rs2307424C>T	C/C	Exon 5, Pro180Pro	138 (0.879)	270 (0.915)	0.216
	C/T		19 (0.121)	25 (0.085)	
NR1I3 rs3003596T>C	T/T	Intron 3	51 (0.325)	100 (0.341)	0.659
	T/C		72 (0.459)	140 (0.478)	
	C/C		34 (0.217)	53 (0.181)	
NR1I3 rs2502815C>T	C/C	Intron 3	88 (0.564)	180 (0.610)	0.173
	C/T		53 (0.340)	100 (0.339)	
	T/T		15 (0.096)	15 (0.051)	

### Variants discovered through targeted sequencing of *NR1I2* and *NR1I3* DNA binding domains (DBD) in 32 HIV/AIDS patients

Targeted sequencing of the DBDs in *NR1I2* [GenBank: AF364606] and *NR1I3* [GenBank: BC069626.1] in 32 HIV/AIDS patients identified a total of 13 genetic variants (Table 3)*.* Only three of these were novel variants discovered in the DBD of *NR1I2 (36726T>C,* in intron 1*; 36857G>A* and *36905C>T,* both in exon 2) (Additional file 2: Figure S1). Using the FANS program, the functional significance of the novel SNPs was predicted. While *NR1I2 36726T>C* change was predicted to be of little functional significance, *NR1I2 36857A* variant was associated with increased binding affinity of SRp40 splicing proteins compared to the *NR1I2 36857G* variant.

**Table 3:** ***NR1I2 *and *NR1I3 *genetic variants in 32 HIV/AIDS patients following targeted sequencing of the *NR1I2 *and *NR1I3 *DNA binding domains**

**SNP ID**	**Location/Functional effect**	**Minor allele**	**Minor allele n (freq)**
NR1I2 rs12721601T>C	Previously reported, Intron 1	C	1 (0.016)
NR1I2 rs59371185G>A	Previously reported, Exon 2, Glu18Lys	A	1 (0.016)
NR1I2 rs12721613C>T	Previously reported, Exon 2, Pro27Ser	T	4 (0.064)
NR1I2 rs1464603C>T	Previously reported, Intron 2, Disrupts HNF1 TFB site	T	1 (0.016)
NR1I2 rs1464602C>T	Previously reported, Intron 2	T	20 (0.313)
NR1I2 rs80320762G>A	Previously reported, Intron 2	A	3 (0.048)
NR1I2 rs12721616C>T	Previously reported, Intron 2	T	14 (0.219)
NR1I2 rs112813596G>A	Previously reported, Intron 3	A	1 (0.016)
NR1I3 rs35205211C>G	Previously reported, Exon 4, Ala86Ala	G	2 (0.032)
NR1I3 rs34161743C>T	Previously reported, Exon 4, Arg97Trp	T	1 (0.016)
NR1I2 36726T>C	Novel, Intron 1	C	5 (0.078)
NR1I2 36857G>A*	Novel, Exon 2	A	1 (0.016)
NR1I2 36905C>T	Novel, Exon 2	T	2 (0.031)

Position is noted according to the *NR1I2* gene sequence [GenBank: AF364606], *Allele change according to the reverse sequence.

### Correlation of *NR1I2* and *NR1I3* variants with plasma efavirenz concentrations, change in treatment regimens and effects of *CYP2B6 516G>T SNP*

**Figure 1:**
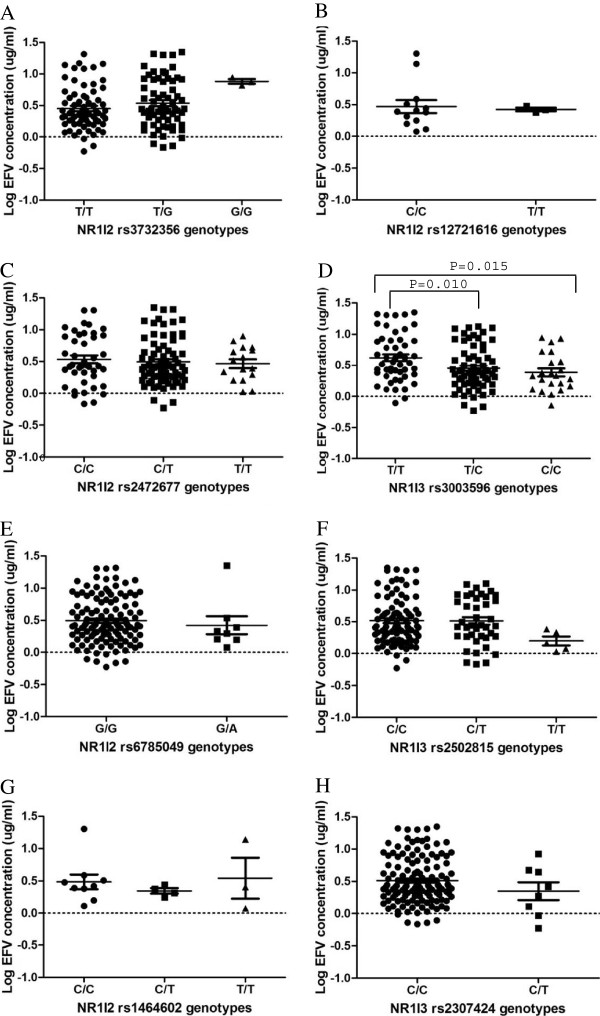
**A-H: Effects of *NR1I2 *and *NR1I3 *variation on plasma efavirenz concentration.** Only significant P-values are indicated.

**Figure 2:**
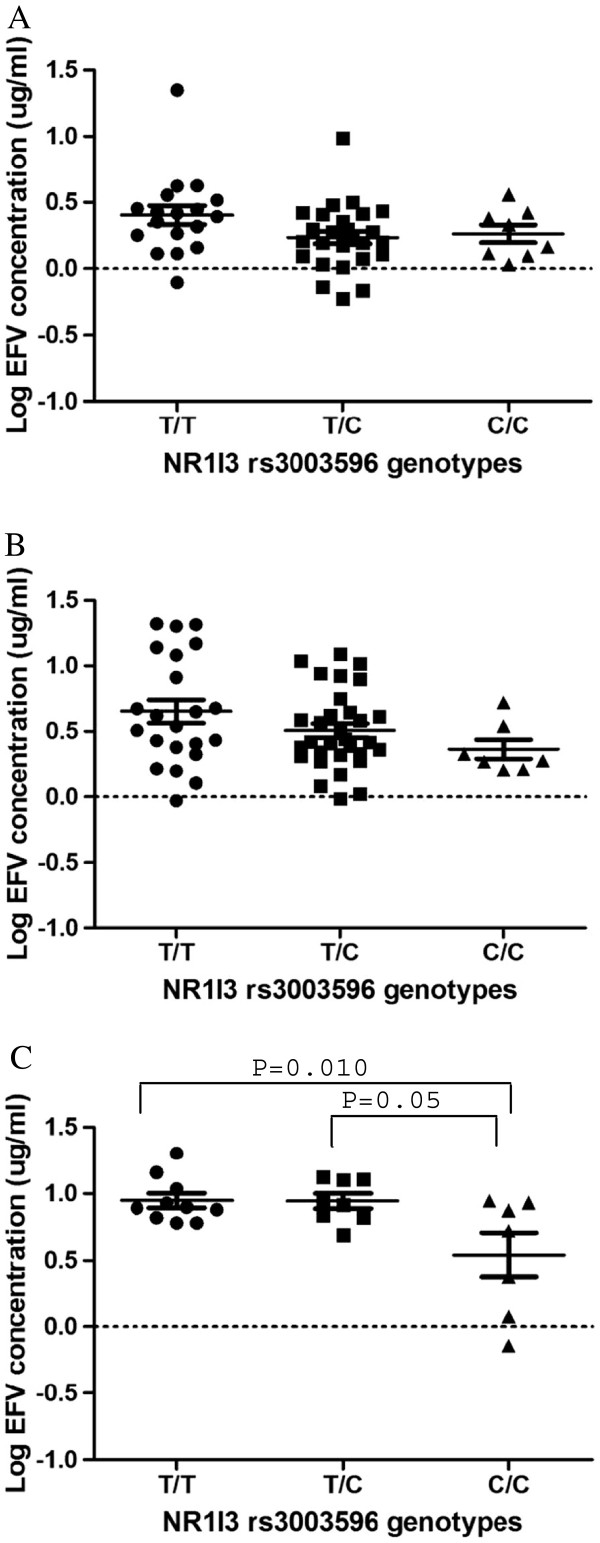
**A-C: Effects of *NR1I3 rs3003596 *and *CYP2B6 G516T *variation on plasma efavirenz concentration.A**) *NR1I3 rs3003596* stratified by *CYP2B6 516G/G*. **B**) Stratified by *CYP2B6 516G/T.***C**) Stratified by *CYP2B6 516T/T.* Only significant P-values are indicated.

The *NR1I3 rs3003596C/C* and *T/C* genotypes were associated with significantly reduced plasma efavirenz concentrations compared to the *NR1I3 rs3003596T/T* genotype with P-values of 0.015 and 0.010, respectively and remained significant after Bonferroni’s correction for multiple comparison tests for the three *NR1I3* SNPs with significant P<0.017 (Figure 1D). Three of the twenty-two (14%) individuals with the *rs3003596C/C* genotype had plasma efavirenz concentrations above 4 μg/mL, while twenty-four of the fifty (48%) individuals with the *rs3003596T/T* genotype had plasma efavirenz concentrations above 4 μg/mL. The trend towards reduced plasma efavirenz levels associated with *NR1I3 rs3003596C/C* and *T/C* genotypes remained despite stratification according to *CYP2B6 G516T* genotypes (Figure 2). CYP2B6 is the main enzyme involved in the metabolism of efavirenz and the *CYP2B6 516T* variant is associated with reduced CYP2B6 activity and inversely, increased plasma efavirenz levels. The effect of the *rs3003596C* variant is clearly demonstrated in Figure 2C where, in the absence of CYP2B6 substantial activity, the variant is associated with significantly decreased efavirenz levels. This points to possible increased *NR1I3* expression in the presence of the *NR1I3 rs3003596C* variant, eliciting its effects through other enzymes that participate in efavirenz metabolism.

Although not statistically significant, *NR1I2 rs3732356G/G* genotype was associated with high plasma efavirenz concentrations while *NR1I3 rs2502815T/T* and *NR1I3 rs2307424C/T* genotypes were associated with reduced plasma efavirenz levels. It was observed that the *NR1I2 rs3732356G/G* genotype was associated with the least likelihood of changing treatment regimens at 3, 6 and 12 months (0%, 0%, 0%), while 4%, 11%, 24% and 3%, 6%, 21% in patients with the *T/T* and *T/G* genotypes, respectively, had their treatment regimens changed (Table 4). However, this finding could have been influenced by the small sample size (n=9) of the *NR1I2 rs3732356G/G* genotype group (Figure 1A). It was also observed that *NR1I3 rs2307424C/T* genotype was associated with the highest number (12%, 20% and 32%) of patients switching from efavirenz-based treatment regimen at all three time intervals, respectively (Table 4).

**Table 4:** Frequency of HIV/AIDS patients changing ART regimens within 3, 6 or 12 months post-initiation of treatment

**Gene**	**ARV regimen or Genotype**	**Treatment initiation (n)**	**3 months**	**6 months**	**12 months**
ARV regimen					
	3TC_TDF_EFV	9	0.11	0.11	0.22
	AZT_3TC_EFV	11	0.00	0.09	0.27
	d4T_3TC_EFV	222	0.03	0.08	0.22
NR1I2					
	rs3732356T/T	156	0.04	0.11	0.24
	rs3732356T/G	117	0.03	0.06	0.21
	rs3732356G/G	9	0.00	0.00	0.00*
	rs2472677C/C	92	0.05	0.06	0.22
	rs2472677C/T	128	0.02	0.11	0.23
	rs2472677T/T	36	0.03	0.11	0.17
	rs6785049G/G	251	0.03	0.08	0.22
	rs6785049G/A	14	0.00	0.07	0.29
NR1I3					
	rs2307424C/C	257	0.02	0.07	0.21
	rs2307424C/T	25	0.12	0.20	0.32
	rs3003596T/T	96	0.04	0.08	0.23
	rs3003596T/C	132	0.03	0.10	0.20
	rs3003596C/C	52	0.02	0.06	0.23
	rs2502815C/C	169	0.02	0.09	0.22
	rs2502815C/T	98	0.05	0.08	0.22
	rs2502815T/T	15	0.00	0.00	0.20

*None of the subjects carrying the *NR1I2 rs3732356G/G* genotype changed their treatment regimen.

Variants associated with reduced plasma efavirenz levels could possibly act through increased transcription of PXR or CAR and therefore increased transcription of downstream DME genes such as *CYP2B6* and *CYP1A2* leading to increased efavirenz clearance through metabolism. Thus, the *NR1I3 rs2307424T* variant may result in the decrease in efavirenz levels to concentrations that are too low for effective viral suppresion, necessitating the change in treatment regimen when physicians note poor viral load decreases. On the other hand, variants associated with increased plasma efavirenz could be acting through decreased transcription of DMEs and are associated with treatment changes due to ADRs. As confirmed from the patient records, the major cause for treatment change in the *NR1I3 rs2307424C/T* genotype group was ineffective viral suppresion, while the major reason in the *NR1I3 rs2307424C/C* genotype group was adverse drug events. Although the amino acid change is synonymous (Pro180Pro), codon usage is known to affect the rate of transcription and to some extent protein folding [22].

### Allele frequencies and their distribution in different populations

The frequencies of the genetic variants detected in *NR1I2* and *NR1I3* among South African subjects were compared to allele frequencies in populations of Caucasian, Asian and Yoruba origin obtained from HapMap or dbSNP (Table 5). Statistically significant differences were observed between the allele frequencies in the South African cohort and the Caucasian, Asian and Yoruba populations for eleven of the twelve SNPs in *NR1I2* and *NR1I3* (P<0.05). The Bantu-speaking South African population showed differences in the distribution of 8 SNPs when compared to the Yoruba population (Table 5), which renders problematic the use of one African population to predict drug response in another.

**Table 5:** Comparison of the allele frequencies between the South Africans and other populations

**SNP ID**	**Minor allele**	**South Africans this study (n=464)**	**YRI HapMap (n=226)**	**CEU HapMap (n=226)**	**HCB HapMap (n=86)**	**Bushman dbSNP (n=3)**
NR1I2 rs3732356T>G	G	0.234	0.254	0.033**	N/A	0.000**
NR1I2 rs2472677C>T	T	0.351	0.356	0.636**	0.544*	N/A
NR1I2 rs6785049G>A	A	0.040	0.000**	0.673**	0.453**	N/A
NR1I3 rs2307424C>T	T	0.047	0.119**	0.336**	0.512**	N/A
NR1I3 rs3003596T>C	C	0.416	0.612**	0.407	0.535	0.500
NR1I3 rs2502815C>T	T	0.230	0.406**	0.248	0.442**	0.500**
		**n=32**^**#**^	**n=226**	**n=226**	**n=86**	
NR1I2 rs12721601T>C	C	0.016	0.008	N/A	N/A	N/A
NR1I2 rs59371185G>A	A	0.016	0.000	N/A	N/A	N/A
NR1I2 rs12721613C>T	T	0.064	0.183*	0.000*	0.000*	N/A
NR1I2 rs1464603C>T	T	0.016	N/A	0.292**	0.325**	0.000
NR1I2 rs1464602C>T	T	0.313	0.186*	0.717**	0.675**	0.667**
NR1I2 rs80320762G>A	A	0.048	0.144*	N/A	N/A	N/A
NR1I2 rs12721616C>T	T	0.219	0.042**	N/A	N/A	N/A
NR1I2 rs112813596G>A	A	0.016	N/A	N/A	N/A	0.000
NR1I3 rs35205211C>G	G	0.032	N/A	N/A	N/A	0.005
NR1I3 rs34161743C>T	T	0.016	N/A	N/A	N/A	0.005
NR1I2 36726T>C^**#**^	C	0.078	N/A	N/A	N/A	N/A
NR1I2 36857G>A^**#**^	A	0.016	N/A	N/A	N/A	N/A
NR1I2 36905C>T^**#**^	T	0.031	N/A	N/A	N/A	N/A

N/A = not available; *allele frequencies statistically significantly different from the South Africans (P<0.05), **allele frequencies statistically significantly different from the South Africans (P<0.001), ^**#**^Sequencing according to the *NR1I2* gene sequence [GenBank: AF364606] with the allele change for *NR1I2 36857G>A* according to the reverse sequence.

### Haplotype analysis

**Figure 3:**
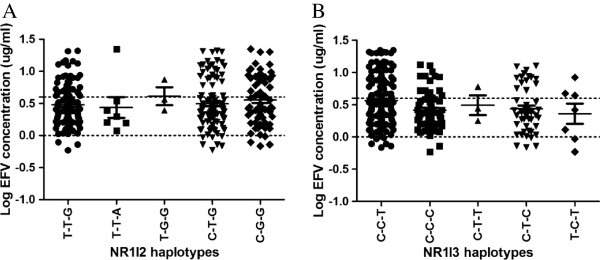
**A-B: Effects of *NR1I2 *and *NR1I3 *haplotypes on plasma efavirenz concentration.** The therapeutic range for efavirenz is shown. *NR1I2 C-T-T* and *NR1I3 T-C-C* haplotypes were only observed once and thus excluded from the analysis.

Haplotype frequencies were compared between the healthy subjects and the HIV/AIDS patients. The haplotype frequencies in *NR1I2* were significantly different between the healthy subjects and HIV/AIDS patients (P=0.015) (Table 6). However, the difference was marginally significant where the haplotype analysis was corrected for multiple testing with significant P<0.017. By observation, the *NR1I2 T-G-G* haplotype (with respect to *rs2472677-rs3732356-rs6785049*), which occurs in about 3% of the HIV/AIDS patients, was associated with efavirenz levels greater than 4 μg/mL, and this may influence treatment regimen change (Figure 3A).

**Table 6:** Comparison of the haplotype frequencies between the healthy subjects and the HIV/AIDS patients

**Haplotypes**	**Healthy subjects n (freq)**	**HIV/AIDS patients n (freq)**	Efavirenz levels (μg/mL)*	**Global p-value**^**#**^
NR1I2 haplotypes [rs2472677-rs3732356-rs6785049]				
C-T-A	7 (0.025)	6 (0.012)	2.13	
C-T-G	109 (0.367)	190 (0.379)	4.00	
C-G-G	64 (0.216)	114 (0.226)	6.69	0.015
T-T-A	16 (0.052)	6 (0.011)	4.94	
T-T-G	96 (0.321)	172 (0.343)	4.23	
T-G-G	6 (0.019)	13 (0.026)	4.55	
NR1I3 haplotypes [rs2307424-rs2502815-rs3003596]				
C-C-T	153 (0.489)	310 (0.530)	4.97	
C-C-C	58 (0.187)	122 (0.208)	3.45	
C-T-T	7 (0.023)	8 (0.013)	3.53	0.424
C-T-C	75 (0.240)	121 (0.207)	3.87	
T-C-T	14 (0.045)	22 (0.037)	4.42	
T-C-C	4 (0.012)	3 (0.005)	1.89	

*Efavirenz levels are for the HIV/AIDS patients only; ^**#**^Global p-value is for comparison of haplotype frequencies between healthy subjects and HIV/AIDS patients.

## Discussion

Many factors, including environmental and genetic factors, determine how individual patients respond to therapeutic drugs. Pharmacogenetics is concerned with understanding individual genetic variability and how it affects response to treatment. Most of the early work has focused on DME genes because of their direct involvement in conversion of drugs and their removal from the body. However, in order to gain a better understanding, variation in the NRs that affect the regulation of DMEs was investigated. This research provides a window into how the variation in the NRs, PXR and CAR, can indirectly affect plasma drug levels and ultimate response to treatment. The report also documents the frequencies of variants for these two genes in the South African population and adds to the growing literature on the genetic characterization of African populations [23,24]. To our knowledge, this is the first report on baseline frequencies for the six SNPs in *NR1I2* and *NR1I3* in a Bantu-speaking South African population.

### Gene variant frequencies in the South African population and comparison to other world populations

The genotype frequencies between the healthy subjects and HIV/AIDS patients differed significantly for the *NR1I2 rs6785049G>A* and *NR1I2 rs3732356T>G* SNPs (P=0.002 and 0.031, respectively). The difference in genotype frequencies for the *NR1I2 rs3732356T>G* SNP between the healthy subjects and HIV/AIDS patients may be explained by the deviation from HWE in the HIV/AIDS patients (P=0.020). Among the HIV/AIDS patients, the *NR1I2 rs6785049A/A* genotype was not observed and the *rs6785049A* variant occurred at a frequency of 2.5% compared to the 7% among healthy subjects. On the other hand, the *NR1I2 rs3732356G* allele frequency was higher among HIV/AIDS patients (48%) compared to healthy subjects (22%). The above findings lead us to postulate that PXR may have a role, not only in the metabolism of drugs, but also indirectly in signalling pathways active during acute stages of infection. Gu *et al.,*[25] showed that NF-kB directly interacts with the DBD of RXR; this in turn prevents RXR binding to its consensus DNA sequences and PXR.

*NR1I2 rs2472677C>T, NR1I2 rs6785049G>A* and *NR1I3 rs2307424C>T* SNPs displayed significant differences in allele frequencies between the South African population and the Caucasian and Asian populations. As a result, therapeutic drugs such as efavirenz that are ligands for NRs, may result in different drug effects in different populations. Similarly, African populations cannot be regarded as homogeneous due to the genetic diversity existing between the sub-populations. For example, three SNPs in *NR1I3* and *NR1I2 rs6785049* showed a significant difference in allele frequencies between the South African population and the Yoruba population from Ibadan, Nigeria.

The SNPs in *NR1I2, rs3732356T>G, rs2472677C>T* and *rs6785049G>A*, as well as in *NR1I3, rs2307424C>T*, *rs3003596T>C* and *rs2502815C>T*, displayed no evidence of LD, which is in contrast to the strong LD for *NR1I3 rs2502815C>T* and *NR1I3 rs2307424C>T* reported in a Caucasian population (D’=1.00; P= 0.01) [26]. This finding is consistent with the weak LD reported in African populations, due to the large degree of genetic diversity in African populations [27].

### Comment on drug discovery and relevance of this knowledge

Sequencing of the *NR1I2* and *NR1I3* DBDs identified several previously characterised SNPs as well as three novel variants in the *NR1I2* DBD. The allele frequency (0.016) of the *NR1I2 52A* allele (*rs59371185*) observed in the 32 sequenced South African individuals was similar to allele frequencies reported in Africans from the Ivory Coast and sub-Saharan Africa [9,28]. The *NR1I2 70C>T* SNP was observed at a frequency of 0.063 in the sequenced samples, but has however, been reported at frequencies of 0.126 and 0.002 in sub-Saharan Africans and Caucasians, respectively [9,28]. *NR1I2 36726T>C* is predicted to be of little significance, since it is present in an intron and is not situated within a GT-AG splice site. Although *NR1I2 36857G>A* and *NR1I2 36905C>T* in exon 2 are both synonymous amino acid substitutions, they may be associated with differential PXR expression due to codon usage [22]. The *NR1I2 36905T* variant was also predicted to affect the structure of PXR. Further analysis (with ESE finder v3.0) revealed that *NR1I2 36857A* affects the binding affinity of the SRp40 splicing protein, which regulates pre-ribosome assembly and transport. Destabilisation of the DBD of NRs is likely to influence the binding of these NRs to promoter regions of target DMEs and possibly alter transcription and expression. Alternatively spliced *NR1I2* mRNA isoforms can differ in their patterns of expression, biological function (either gain- or loss-of-function), activation of target genes like DMEs, DNA binding and tissue-specific expression [29], which may contribute to inter-individual variability in *NR1I2* expression and ultimately efavirenz metabolism.

### Implications for disease or drug treatment and possible development of diagnostic tools

A significantly low average plasma efavirenz concentration was observed among patients with the *NR1I3 rs3003596C/C* and *T/C* genotypes compared to patients with the *rs3003596T/T* genotype (P=0.015 and P=0.010, respectively), using a dominant genetic model. The low efavirenz concentrations may point to possible functional effects of the change on CAR, expression or activity, and regulation of multiple target genes encoding DMEs. Efavirenz induces CYP2B6 activity primarily through CAR and genetic variation in *NR1I3* may therefore significantly influence efavirenz plasma concentrations via the induction of CYP2B6. Due to the availability of resources in Western and Asian countries, these populations have been well studied in comparison to the African populations [30,31]. Thus, most drug-discovery and development is premised on Caucasian and Asian populations, and as a result, new drugs may make it to the market without having been exposed to most of the genetic variability within African populations. The effect of this variability only becomes obvious when adverse drug events are noticed [30]. For example, the ART guidelines initially established by the World Health Organization, on which the South African guidelines are modelled, was based on clinical data obtained primarily from Caucasian and Asian individuals. Understanding the consequence of genetic variation in *NR1I2* and *NR1I3* adds to the pharmacogenetics knowledge and improves the move towards personalised medication. This data, taken together with variation in DMEs that metabolise efavirenz (e.g. CYP2B6 and CYP2A6), may aid in the designing of appropriate genotyping assays that could prove useful in individualized efavirenz dosing regimens.

## Conclusion

The finding of novel variants in *NR1I2* after sequencing a very small portion of the DBD is further testimony that there is still “missing” genetic heritability that can be discovered when sequencing as many African genomes as possible. In addition, our data highlights the role (though indirect) that variation in NR genes can play in drug treatment response.

## Competing interests

All authors declare that they have no competing interest.

## Authors’ contributions

MS carried out 40% the molecular genetic studies and drafted the manuscript. HW carried out 60% of the molecular genetic studies. YR and PS both carried out the LC/MS/MS analysis of plasma efavirenz concentration. RR contributed towards reagents and equipment. CD conceived of the study, designed, coordinated the study, assisted with statistical data analysis and helped to draft the manuscript and approved the final version. All authors read and approved the final manuscript.

## Pre-publication history

The pre-publication history for this paper can be accessed here:

http://www.biomedcentral.com/1471-2350/13/112/prepub

## Supplementary Material

Additional file 1 **Table S1.** PCR-RFLP and SNaPshot genotyping of SNPs in *NR1I2* and *NR1I3* and sequencing of *NR1I2* and *NR1I3* DNA binding domains. Click here for file

Additional file 2 **Figure S1.** Chromatograms of the novel variants at position *NR1I2 36726T>C* in intron 1, *NR1I2 36857G>A* (allele change according to the reverse sequence) in exon 2 and *NR1I2 36905C>T* in exon 2. Click here for file
