# Supplementary material for: PXR and CAR single nucleotide polymorphisms influence plasma efavirenz levels in South African HIV/AIDS patients
Source: BMC Med Genet. 2012 Nov 22;13:112. doi: 10.1186/1471-2350-13-112 (PMC3523080; doi:10.1186/1471-2350-13-112)
Supplement: Additional file 1 — Table S1. PCR-RFLP and SNaPshot genotyping of SNPs in NR1I2 and NR1I3 and sequencing of NR1I2 and NR1I3 DNA binding domains. [file 1471-2350-13-112-S1.doc]

Supplementary Table S1: PCR-RFLP and SNaPshot genotyping of SNPs in *NR1I2* and *NR1I3* and sequencing of *NR1I2* and *NR1I3* DNA binding domains.

| **SNP ID** | **Primer Sequence (5'-3')** | **T_a_ (°C)** | **Amplicon Size (bp)** | **Genotyping Method** |
| --- | --- | --- | --- | --- |
| NR1I2 rs2472677C>T | F: TGTGTGGGCATAGGTTTTCA | 62 | 665 | RFLP with *Hpy*188I  C:553,66,46bp  T: 619,66bp |
|  | R: AAGCATATTACTGGTGGTTGGT |  |  |  |
| NR1I2 rs3732356T>G | F: GCACGGCTCTGAGTAAGGAC | 50 | 320 | SNaPshot |
|  | R: CCCCCAGATTCACACTCCTA |  |  |  |
|  | SNaPshot: CATCTCCACACAAGCATGC |  |  |  |
| NR1I3 rs2502815C>T | F: TGAAGACCCCTCCCTCTACA | 62 | 434 | SNaPshot |
|  | R: TGTCCGGATCAGCTCTTCTT |  |  |  |
|  | SNaPshot: GGAATTCCCCCCACTGCCCCATCCTT |  |  |  |
| NR1I3 rs3003596T>C | F: TGCTTTTTCCATATCACACGA | 50 | 224 | SNaPshot |
|  | R: ACCCAGCCCAATCACATTT |  |  |  |
|  | SNaPshot: tcatatgacTTCTGCAAAAGATCCAAGATCA | |  |  |
| NR1I3 rs2307424C>T | F: TGCCTCTGGTCACACACTTC | 58 | 156 | SNaPshot |
|  | R: GTGACTTTTCGGGGTGGATA |  |  |  |
|  | SNaPshot: gtaatacaAGTCATCAAGTTTACTA AGGACCTGCC | |  |  |
| NR1I2 rs6785049G>A | F: TGGATGCCAAGCTCAGTGG | 50 | 194 | SNaPshot |
|  | R: CAGCAGCCATCCCATAATCC |  |  |  |
|  | SNaPshot: GCCATCCTCCCTCTTCCTCTC |  |  |  |
| NR1I2 DBD (Exon 2) | F: AACAATTCCAACCCCCATTC | 58 | 542 | Sequencing |
|  | R: TCTGGGATATAAATGGCTCCC |  |  |  |
| NR1I2 DBD (Exon 3) | F: ACTCCCACCTACACCCTTCC | 58 | 504 | Sequencing |
|  | R: CTCCCTCCATCTCCCAGAG |  |  |  |
| NR1I3 DBD (Exon 4) | F: TGAAGACCCCTCCCTCTACA | 56 | 544 | Sequencing |
|  | R: TTTGGGTGCCCTTTTAGTTG |  |  |  |
