# Supplementary material for: PXR and CAR single nucleotide polymorphisms influence plasma efavirenz levels in South African HIV/AIDS patients
Source: BMC Med Genet. 2012 Nov 22;13:112. doi: 10.1186/1471-2350-13-112 (PMC3523080; doi:10.1186/1471-2350-13-112)
Supplement: Additional file 2 — Figure S1. Chromatograms of the novel variants at position NR1I2 36726T>C in intron 1, NR1I2 36857G>A (allele change according to the reverse sequence) in exon 2 and NR1I2 36905C>T in exon 2. [file 1471-2350-13-112-S2.doc]

A


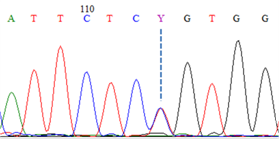


B


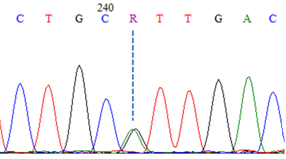


C


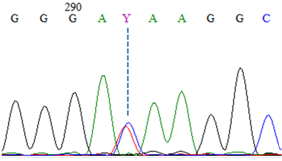


**Supplementary Figure S1:** Chromatograms of the novel variants at position *NR1I2 36726T>C* in intron 1, *NR1I2 36857G>A* (allele change according to the reverse sequence) in exon 2 and *NR1I2 36905C>T* in exon 2.
